# A Weed-Derived Hierarchical Porous Carbon with a Large Specific Surface Area for Efficient Dye and Antibiotic Removal

**DOI:** 10.3390/ijms23116146

**Published:** 2022-05-30

**Authors:** Dadong Liang, Xingyi Tian, Yupeng Zhang, Guanya Zhu, Qiang Gao, Junbo Liu, Xiaoxiao Yu

**Affiliations:** 1 College of Resource and Environment, Jilin Agricultural University, Changchun 130118, China; liangdadong@jlau.edu.cn (D.L.); tianxingyi222@163.com (X.T.); zhangyupeng1213@163.com (Y.Z.); zhuguanyacyy99@163.com (G.Z.); gyt199962@163.com (Q.G.); 2 Key Laboratory of Straw Comprehensive Utilization and Black Soil Conservation, The Ministry of Education, Jilin Agricultural University, Changchun 130118, China; yx8751@sina.com

**Keywords:** *Abutilon theophrasti* medicus calyx, porous carbon, adsorption, dye, antibiotic

## Abstract

Adsorption is an economical and efficient method for wastewater treatment, and its advantages are closely related to adsorbents. Herein, the *Abutilon theophrasti* medicus calyx (AC) was used as the precursor for producing the porous carbon adsorbent (PCAC). PCAC was prepared through carbonization and chemical activation. The product activated by potassium hydroxide exhibited a larger specific surface area, more mesopores, and a higher adsorption capacity than the product activated by sodium hydroxide. PCAC was used for adsorbing rhodamine B (RhB) and chloramphenicol (CAP) from water. Three adsorption kinetic models (the pseudo-first-order, pseudo-second-order, and intra-particle diffusion models), four adsorption isotherm models (the Langmuir, Freundlich, Sips, and Redlich–Peterson models), and thermodynamic equations were used to investigate adsorption processes. The pseudo-second kinetic and Sips isotherm models fit the experimental data well. The adsorption mechanism and the reusability of PCAC were also investigated. PCAC exhibited a large specific surface area. The maximum adsorption capacities (1883.3 mg g^−1^ for RhB and 1375.3 mg g^−1^ for CAP) of PCAC are higher than most adsorbents. Additionally, in the fixed bed experiments, PCAC exhibited good performance for the removal of RhB. These results indicated that PCAC was an adsorbent with the advantages of low-cost, a large specific surface area, and high performance.

## 1. Introduction

During the processes of industrial production and human life, large volumes of wastewater containing threatening pollutants such as antibiotics and dyes have been produced. These pollutants should be removed from water to avoid a huge health hazard to human beings, animals, and plants [1,2,3]. So far, various techniques and methods of wastewater treatment such as nanotechnology [4], biotechnology [5], molecularly imprinted technology [6,7,8,9], fuel cell technology [10], advanced oxidation [11], ultrafiltration [12], and adsorption have been developed. Among them, adsorption is promising because of its advantages, which include high efficiency, ease of operation, and a low cost. So far, some important cost-efficient adsorbents including clays [13] and porous carbons [14,15] have been exploited for water and wastewater treatment. Moreover, other functional materials such as zeolites [16], metal–organic frameworks [17,18], hydrogels [19], graphene [20], and their composites [21,22,23,24] may also exhibit excellent adsorption performance for the removal of pollutants.

In recent years, biomass-derived porous carbons have attracted much attention in the field of wastewater treatment. Various biomasses such as grasses, leaves, hyphae, and agricultural wastes have been used as the precursors for producing porous carbon adsorbents [25,26,27,28,29]. Through being heated under anaerobic conditions, these lignocellulosic biomasses were converted into biochars [29]. Usually, the produced biochars exhibited low specific surface areas and poor adsorption abilities. Thus, the biomasses or biochars should be treated in the presence of chemical activators such as zinc chloride (ZnCl_2_), potassium hydroxide (KOH), and sodium hydroxide (NaOH). For example, ZnCl_2_ may act as a dehydration agent, and the utilization of ZnCl_2_ may restrain the formation of tar and facilitate the formation of porous structures of carbon products [30,31]. KOH is another effective chemical activator. KOH may react with biochar. Moreover, through a series of chemical reactions (Equations (S1)–(S5), in supporting information (SI)), potassium carbonate (K_2_CO_3_), potassium oxide (K_2_O), kalium (K), water (H_2_O), and carbon dioxide (CO_2_) are produced. K_2_CO_3_ and K_2_O may further etch biochar through redox reactions. The formation of H_2_O and CO_2_ has a positive contribution to the development of porous structures. K atoms may intercalate into the carbon lattices, resulting in the expansion of the lattices and the formation of porous structures [32]. In addition, the biochars may also be etched by NaOH through a series of chemical reactions (Equations (S6)–(S8), in SI) [33]. During such chemical activation processes, a large number of micro- and mesopores occurred on the surfaces of carbons, which enabled the carbon products to exhibit large specific surface areas and high adsorption capacities toward adsorbates. Indeed, chemical activation processes increased the costs of carbon products. However, when these porous carbon adsorbents were used in some relatively expensive devices such as chromatographic columns, household water filters, and portable water filters, they were competitive with other materials because of the extremely low cost of biomass precursors and the high performance of porous carbon products.

The choice of biomass precursors is a crucial problem of porous carbon preparation. There are no two biomass precursors which have exactly the same components and natural structures. Thus, the porous carbons derived from different precursors may exhibit different properties and performances. As a result, much effort has been devoted to biomass screening for producing porous carbon materials with outstanding properties (such as large special surface area and high porosity) and an excellent performance in the removal of pollutants [34,35].

*Abutilon theophrasti* medicus is an annual weed which is widely spread across Asia, Europe, America, and other parts of the world [36]. It is prevalent and troublesome in orchards, vegetable farms, and cropping fields [37,38]. The competition of *Abutilon theophrasti* medicus may cause an obvious loss of yields of cotton, corn, sugar beet, and other crops. It can be erased by herbicides [39]. However, the abuse of herbicides may potentially cause environmental pollution. In our opinion, it is meaningful to use this threatening plant to produce useful porous carbon materials. On one hand, the cost of the weed is close to zero. On the other hand, the utilization of the weed may decrease its threat to the growth of economical crops.

Herein, we reported the preparation of a hierarchical porous carbon using the *Abutilon theophrasti* medicus calyx (AC) as the precursor through the processes of carbonization and subsequent chemical activation. After AC was carbonized, the produced biochar (CAC) was activated by KOH or NaOH for creating micro- and mesopores in the biochar. The effects of chemical activation conditions (chemical activator species, heating temperature, heating time, ratio of activator to carbon) on the adsorption abilities of products were explored. The resulting porous carbon derived from AC (PCAC) was used as the adsorbent for removing the representative dye (rhodamine B, RhB) and antibiotic (chloramphenicol, CAP) from water. Three non-linear adsorption kinetic models (the pseudo-first-order, pseudo-second-order, and intra-particle diffusion models) and four non-linear adsorption isotherm models (the Langmuir, Freundlich, Sips, and Redlich-Peterson (R-P) models) were used to fit the adsorption experimental data. The corresponding adsorption mechanism and the reusability of PCAC were investigated. Moreover, the fixed-bed column experiments were carried out for evaluating the adsorption performance of PCAC.

## 2. Results and Discussion

### 2.1. Preparation of Porous Carbon

RhB, a representative dye with high chemical stability, was used as the model adsorbate for evaluating the adsorption abilities of products. After AC was carbonized, the produced biochar exhibited poor adsorption ability. The RhB adsorption capacities of biochar were in the range of 10–50 mg g^−1^ when the samples of biochar (20 mg) were added into the RhB solutions (200 mL), with the initial concentration ranging from 100 to 400 mg L^−1^. In these experiments, the pH values of RhB solutions were not adjusted. The N_2_ adsorption experiment indicated that the Brunauer–Emmer–Teller (BET) specific surface area of the biochar was only 0.474 m^2^ g^−1^. Thus, the low adsorption ability of biochar may be largely due to its low surface area. Next, the biochar was activated through being mixed with chemical activators (KOH or NaOH) and heated under N_2_ atmosphere. The effects of the species of activator, heating temperature, heating time, and the activator-to-carbon ratio on the RhB adsorption capacities of products were explored.

**Figure 1:**
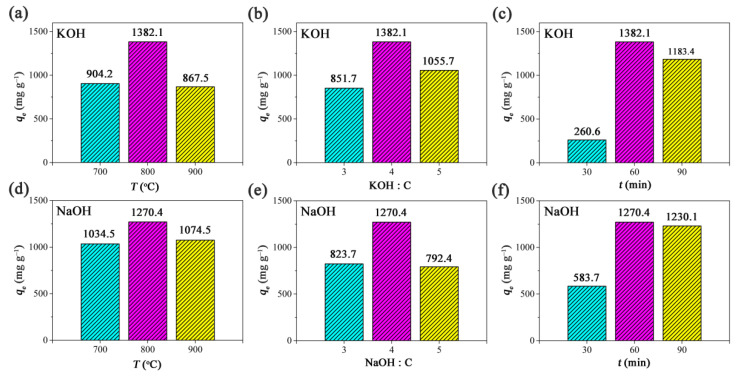
The relationship between the RhB adsorption capacities of porous carbon products and the activation conditions with (**a**–**c**) KOH and (**d**–**f**) NaOH used as the activators, respectively. Conditions: adsorbent, 20 mg; solution volume, 200 mL; initial concentration, 400 mg L^−1^; temperature, 30 °C. The pH values of RhB solutions were not adjusted.

Initially, KOH was used as the activator. The activation temperature was in the range of 700–900 °C, the ratio of activator to carbon was in the range of 3:1–5:1, and the activation time was in the range of 30–90 min. As shown in Figure 1a, when the temperature increased from 700 to 800 °C, the RhB adsorption capacity increased from 904.2 to 1382.1 mg g^−1^. This indicates that the adsorption ability of the product was effectively improved though the chemical reactions between carbon and KOH, and a higher reaction temperature may result in a higher reaction rate. However, when the temperature further increased from 800 to 900 °C, the RhB adsorption capacity decreased from 1382.1 to 867.5 mg g^−1^. The decrement in adsorption capacity may be due to the collapse of the porous structure, which usually occurs under extremely harsh conditions. This situation may be described as an overreaction. Thus, the optimal activation temperature should be 800 °C because the highest adsorption capacity was achieved. Similarly, as shown in Figure 1b,c, one can find that the optimal ratio of KOH to carbon should be 4:1, and the optimal heating time should be 60 min. Under optimal conditions, the yield of PCAC was found to be in the range of 30–40%.

Then, NaOH was used for replacing KOH as the activator. As shown in Figure 1d–f; when NaOH was used, the optimal activation temperature was 800 °C, the optimal ratio of NaOH to carbon was 4:1, and the optimal heating time was 60 min. The yield of porous carbon was in the range of 30–40%, when the biochar was activated by NaOH. It was found that the porous carbon product activated by KOH exhibited a higher RhB adsorption capacity (1382.7 mg g^−1^) than that (1270.4 mg g^−1^) of the product activated by NaOH. In the following adsorption experiments, the product activated by KOH was used as the adsorbent because of its high adsorption ability.

### 2.2. Characterization of Adsorbent

PCAC activated by KOH was characterized by N_2_ adsorption/desorption measurements at 77 K in the relatively pressure (*P*/*P*_0_) range of 0–0.99. The specific surface area was 4023.16 m^2^ g^−1^, obtained through analyzing the isotherm using the multi-point BET method in a linear *P*/*P*_0_ range of 0–0.25. The micropore volume, micropore area, and external surface area were 0.44 cm^3^ g^−1^, 1045.31 m^2^ g^−1^, and 2977.85 m^2^ g^−1^, respectively, as calculated from the N_2_ adsorption isotherm through the t-plot method of micropore analysis. Moreover, the surface area and the pore volume were 2426.42 m^2^ g^−1^ and 2.13 dm^3^ g^−1^, respectively, as determined by the density functional theory (DFT) method. The average pore diameter was 2.38 nm.

**Figure 2:**
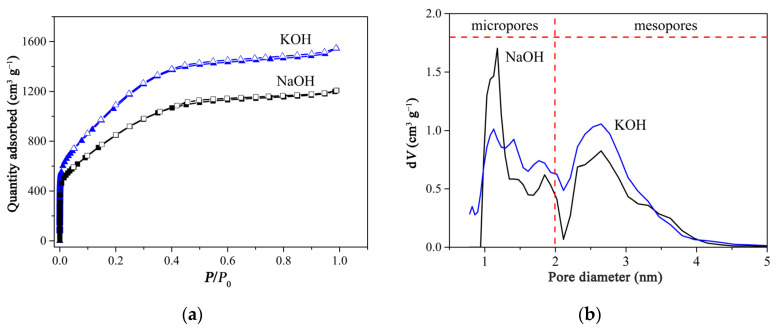
(**a**) N_2_ adsorption/desorption isotherms and (**b**) pore size distribution curves of porous carbon products activated by KOH and NaOH, respectively.

The product activated by NaOH was also characterized, and the BET specific surface area was 3113.44 m^2^ g^−1^ through analyzing the isotherms in the linear *P*/*P*_0_ range of 0–0.25. The micropore volume, micropore area, and external surface area were 0.36 cm^3^ g^−1^, 835.03 m^2^ g^−1^ and 2278.41 m^2^ g^−1^, respectively, calculated through micropore analysis (t-Plot method). The surface area and the pore volume were 1807.50 m^2^ g^−1^ and 1.66 dm^3^ g^−1^, respectively, determined by the DFT method. The average pore diameter was 2.39 nm. In sum, both KOH and NaOH may create micro- and mesopores on the surface of carbon. However, as shown in the pore size distribution curves (Figure 2b), KOH created more mesopores, and NaOH created more micropores.

**Figure 3:**
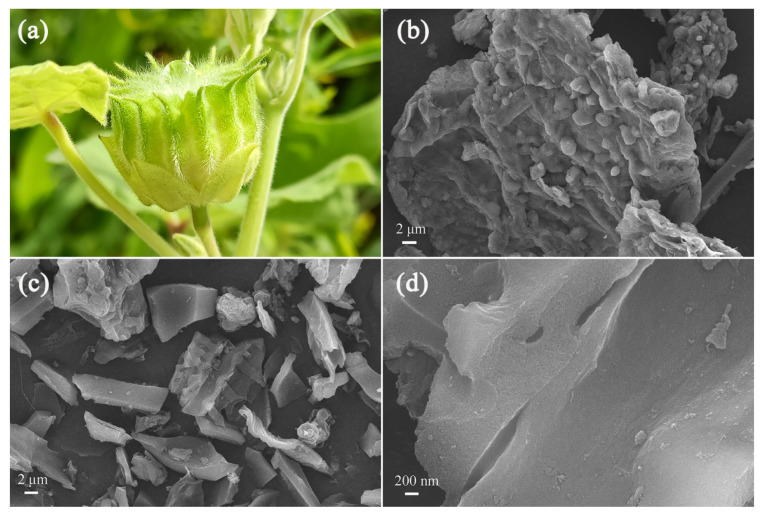
(**a**) The photograph of AC; the SEM images of (**b**) AC fragments and (**c**,**d**) PCAC.

After AC (Figure 3a) was crushed, the fragments exhibited sheet-like morphologies (Figure 3b), which may facilitate contact between lignocellulose and chemical activators and enable PCAC to exhibit a large specific surface area and a high porosity. As shown in Figure 3c,d, through chemical activation, the surfaces of carbon were etched, and the sheet-like morphologies were maintained to some degree. According to the SEM images (Figure S1 in SI), the average particle size of PCAC was approximately 9.0 µm.

**Figure 4:**
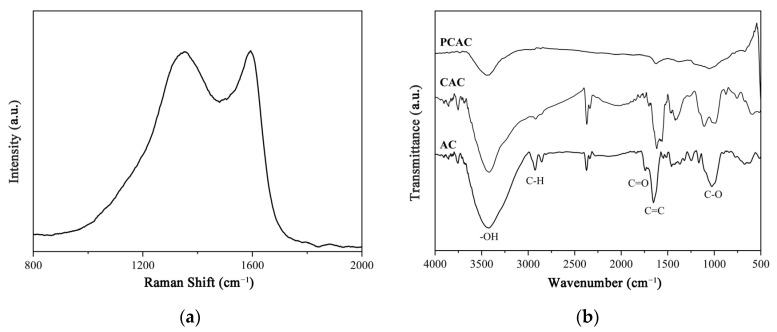
(**a**) Raman pattern of PCAC; (**b**) IR spectra of biomass, biochar, and porous carbon.

The results of the elemental analysis showed that the mass fractions of C, H, N, S, and O elements in PCAC were 86.04, 0.69, 0.17, 0.00, and 6.59, respectively. This indicates that the main component of PCAC was carbon. In the Raman pattern of PCAC (Figure 4a), there are two characteristic peaks at 1360 (D-band) and 1580 cm^−1^ (G-band), respectively, which may be assigned to the disordered carbon and the crystalline carbon, respectively. The intensity ratio of *I_D_* to *I_G_* was 0.99. This demonstrates that PCAC contains both defective graphitic structures and graphitic layers [40].

The samples of biomass, biochar, and porous carbon were characterized by IR (Figure 4b). In the IR spectrum of AC, the peaks at 3423, 2927, 1743, 1634, and 1029 cm^−1^ may be attributed to the -OH, C-H, C=O, C=C, and C-O stretching vibrations, respectively. This indicates that the components of AC are similar to other biomasses, which are mainly composed of lignin, hemicellulose, and cellulose [41,42]. The IR spectrum of CAC is similar to that of AC. However, in the spectrum of PCAC, the intensities of these bands obviously decreased, which demonstrated that partial functional groups had been destroyed by the activator.

Moreover, the types of elements and functional groups in the surface of PCAC were investigated by XPS (Figure S2 in SI). On the sample surface, C and O elements were detected, and no N element was found. The high-resolution C 1 s spectrum may be fit into five individual peaks, which were attributed to the O-C=O (289.6 eV), C=O (286.7 eV), C-O (285.6 eV), C=C (284.9 eV), and C-C (284.5 eV) groups, respectively. The O 1 s spectrum may be fit to three peaks, which were attributed to the -OH (534.0 eV), -C-O (533.0 eV), and -C=O (532.0 eV) groups, respectively [43,44]. These results are in agreement with the IR data.

### 2.3. Batch Adsorption Experiments

#### 2.3.1. Adsorption Kinetics

**Figure 5:**
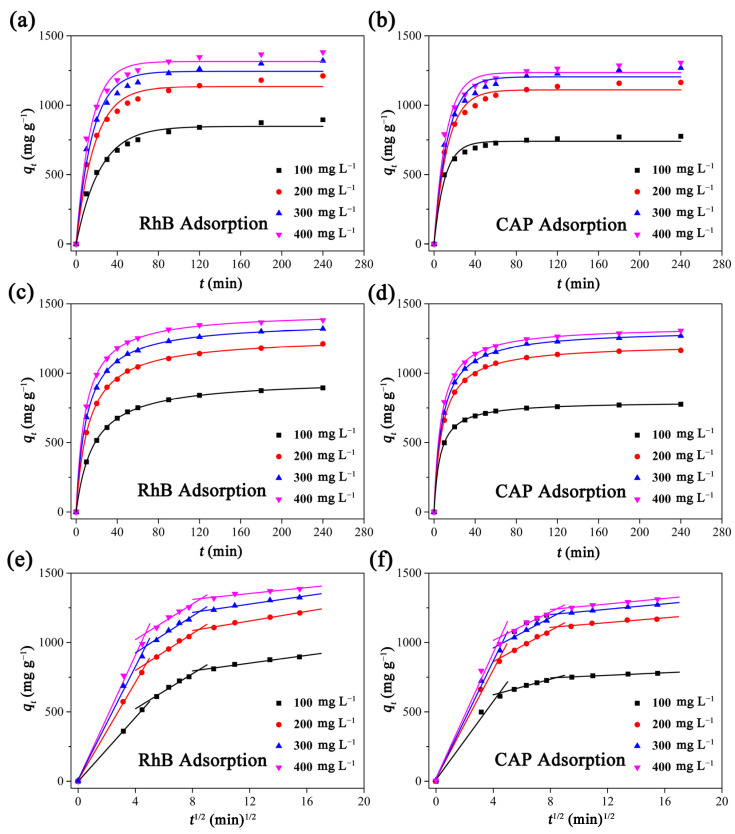
The (**a**,**b**) nonlinear pseudo-first order, (**c**,**d**) pseudo-second order, and (**e**,**f**) intra-particle diffusion kinetic models for the adsorption of PCAC toward RhB and CAP, respectively. Conditions: adsorbent, 20 mg; solution volume, 200 mL; initial concentration, 100–400 mg L^−1^; temperature, 30 °C. The pH values of solutions were not adjusted.

As shown in Figure 5, PCAC could rapidly and effectively adsorb RhB and CAP from their aqueous solutions. For example, in the initial 30 min, the RhB adsorption capacities of PCAC reached 608.4, 898.6, 1015.7, and 1105.7 mg g^−1^, respectively, when the initial concentrations of RhB were 100, 200, 300, and 400 mg L^−1^, respectively (Figure 5a). Then, the RhB adsorption capacities increased gradually and reached 894.1, 1211.2, 1320.2, and 1382.1 mg g^−1^, respectively, at the adsorption equilibrium. A similar trend was observed in the adsorption processes of CAP on PCAC (Figure 5b).

The pseudo-first-order and the pseudo-second-order models, two classic adsorption kinetic models, were used to fit the experimental data, respectively. For the RhB adsorption of PCAC, the curves of the pseudo-second-order model matched the relationship between the equilibrium adsorption capacities and the equilibrium concentrations better than the curves of the pseudo-first-order model. As shown in Table S1 in SI, the correlation coefficient (*R*^2^) values of the pseudo-second-order model were all 0.9998, which are higher than that (0.9773–0.9837) of the pseudo-first-order model. At the same time, the values of *SSE* of the pseudo-second-order model were in the range of 11.69–28.45, which are lower than that (1158.38–3350.42) of the pseudo-first-order model. This demonstrates that the pseudo-second-order model fits the experimental data better. For the CAP adsorption of PCAC, the pseudo-second-order model was also confirmed to be better than the pseudo-first-order model, because of the higher *R*^2^ values, lower values of *SSE*, and better match between the curve and the experimental data (Figure 5c,d). Moreover, the relationships between the adsorption capacities and the contact time were also analyzed using the intra-particle diffusion model (Figure 5e,f). The adsorption of PCAC toward RhB (or CAP) may be divided into three kinetics stages.

#### 2.3.2. Adsorption Isotherms

**Figure 6:**
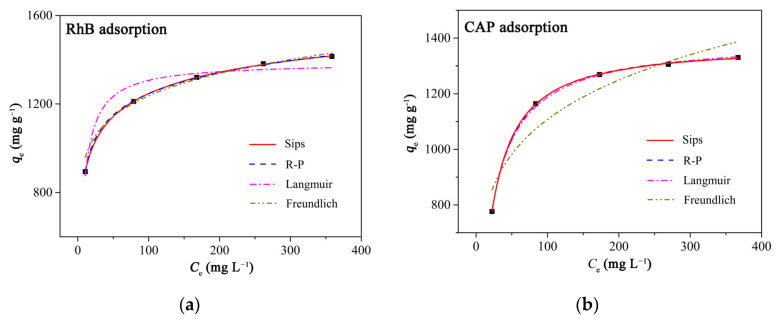
The relationships between the equilibrium adsorption capacities of (**a**) RhB and (**b**) CAP on PCAC and the initial adsorbate concentrations fit the Langmuir, Freundlich, Temkin, R-P, and Sips isotherm models, respectively. Conditions: adsorbent, 20 mg; solution volume, 200 mL; initial concentration, 100–500 mg L^−1^; Temperature, 30 °C. The pH values of RhB solutions were not adjusted.

Two two-parameter adsorption isotherm models (Langmuir and Freundlich models) and two three-parameter adsorption isotherm models (Sips and R-P models) were used to fit the experimental adsorption data in order to further investigate the adsorption processes (Figure 6 and Table 1). The results showed that when these models were used to analyze the results of RhB adsorption of PCAC, the *R*^2^ values of the Langmuir, Freundlich, R-P, and Sips models were 0.9272, 0.9855, 0.9998, and 0.9992, respectively, while the values of *SSE* of these models were 3232.34, 1924.38, 9.00, and 32.55, respectively. For the adsorption of CAP on PCAC, the *R*^2^ values of the Langmuir, Freundlich, Sips, and R-P models were 0.9988, 0.8705, 0.9998, and 0.9995, respectively, while the values of *RSS* of these models were 58.80, 6773.32, 10.22, and 22.26, respectively. As a result, the Sips model fits the adsorption of PCAC toward the adsorbates well because the highest values of *R*^2^ and the lowest values of *SSE* were achieved.

On the basis of the Sips model, the adsorption processes of PCAC toward RhB (or CAP) may be divided into two stages. At lower concentrations of adsorbates, the multilayer adsorption occurred on the surfaces of PCAC; at higher concentrations of adsorbates, the monolayer adsorption occurred on the surfaces of PCAC. According to the Sips model, the maximum adsorption capacities of PCAC towards RhB and CAP were calculated to be 1883.3 and 1375.3 mg g^−1^, respectively. As shown in Table 2 and Table 3, PCAC exhibited higher adsorption capacities toward RhB and CAP than most adsorbents including some graphene-contained materials, which indicates that PCAC is a highly effective adsorbent with low cost.

#### 2.3.3. Adsorption Thermodynamics

The effect of environmental temperature on the adsorption performances of PCAC toward RhB and CAP was explored. The RhB adsorption capacities of PCAC increased along with the increment in temperature. When temperature was 30, 40, and 50 °C, the adsorption capacities were 1382.1, 1405.2, and 1429.2 mg g^−1^, respectively (Table 4). Based on Equations (9)–(11), the values of Δ*H* and Δ*S* were confirmed to be 2.10 kJ mol^−1^ and 20.76 J mol^−1^ K^−1^, respectively, according to the slope and the intercept of the linear relationship, respectively. The positive value of Δ*H* indicates that the total processes of RhB adsorption on PCAC was endothermic. In an aqueous solution, RhB molecules were surrounded by water molecules. When RhB molecules were adsorbed by PCAC, the dehydration process happened. The positive value of Δ*H* may mean that the energy consumed in the dehydration process was higher than the energy released in the process of attaching RhB molecules onto the surface of PCAC [61]. The value is lower than 40 kJ mol^−1^, which is the characteristic of physisorption. The positive value of Δ*S* means that the adsorbent–adsorbate system is more disordered during the adsorption processes. The values of Δ*G* were −4.19, −4.40, and −4.61 kJ mol^−1^, respectively, when temperature gradually increased. The negative values of Δ*G* show the spontaneous character of the adsorption processes. The change of Δ*G* along with the increment in temperature indicates that the adsorption processes may be controlled by thermal energy to some degree [62]. Compared with the RhB adsorption of PCAC, there are some differences in the CAP adsorption of PCAC. The CAP adsorption capacities of PCAC slightly decreased along with the increment in temperature. The values of Δ*H* and Δ*S* were −2.12 kJ mol^−1^ and 6.12 J mol^−1^, respectively. The values of Δ*G* decreased from −3.98 to −4.10 kJ mol^−1^ along with the increment in temperature.

#### 2.3.4. Adsorption Mechanism

**Figure 7:**
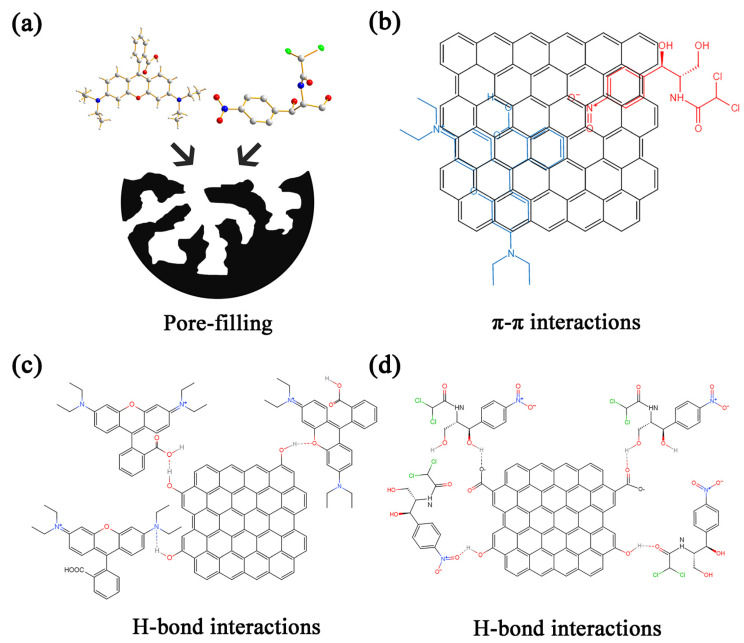
Adsorption mechanism of RhB and CAP on PCAC: (**a**) pore-filling, (**b**) π-π interactions, and (**c**,**d**) H-bond interactions.

PCAC can effectively adsorbed RhB and CAP, which may be due to several reasons (Figure 7). Firstly, the N_2_ adsorption experiment indicates that PCAC is a hierarchical porous material which contains abundant micro- and mesopores (Figure 2b), and the molecules of RhB and CAP may diffuse into these pores and be fixed inside the pores. Secondly, the Raman spectrum (Figure 4a) proved the presence of graphene layers in PCAC. Thus, RhB and CAP molecules may be attached on the surface of the adsorbent through π-π interactions. Thirdly, as shown in the IR and XRS patterns (Figure 4b and Figure S2), in PCAC, there are -OH, C-O, and C=O bonds that may interact with RhB and CAP through H-bond interactions.

In order to explore the potential electrostatic interaction between adsorbent and adsorbate, the point of zero charge (PZC) of PCAC was investigated, and the pH_PZC_ was confirmed to be about 6.5 (Figure S3 in SI). This indicates that, when the pH of the solution was lower than 6.5, the surface of adsorbent exhibited positive characteristic. In contrast, when the pH of the solution was higher than 6.5, the surface of the adsorbent had negative charges.

Furthermore, the adsorption of PCAC toward RhB was carried out with the initial pH of the solution ranging from 3 to 11. RhB molecule contains both the carboxyl group and the amino group. In an acid aqueous solution, RhB exhibits a cationic characteristic. Therefore, when the pH of solution was lower than 6.5, there was electrostatic repulsion between RhB and PCAC. Even so, the PCAC exhibited high RhB adsorption capacities, which demonstrates that the electrostatic interaction is not crucial during adsorption processes. The RhB adsorption capacities of PCAC slightly decreased along with the increase in pH (Figure S3 in SI). On possible reason is that when the pH of RhB solution increased, the gradual aggregation of adjacent RhB molecules happened through the interaction between the carboxyl groups and the amino groups [50]. The increased sizes made it difficult for RhB molecules to diffuse into some relatively narrow pores of PCAC. In sum, no obvious electrostatic interaction was found between PCAC and the adsorbate.

#### 2.3.5. Effect of Ionic Strength

**Figure 8:**
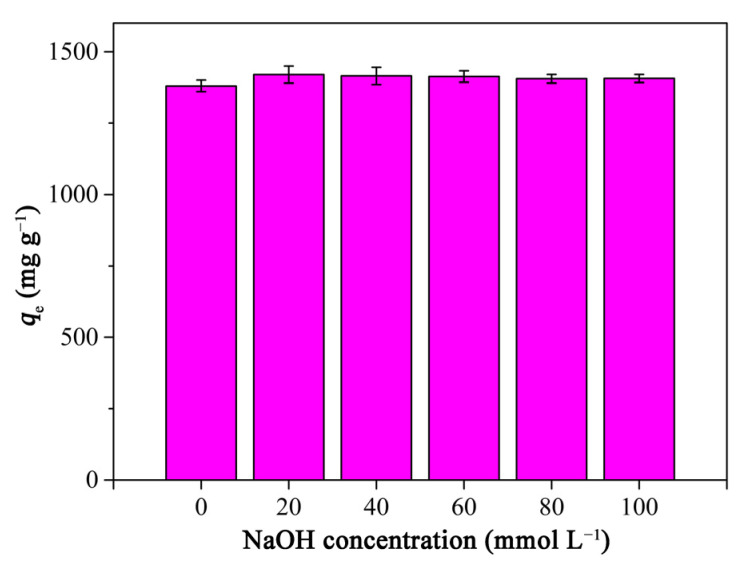
Effects of ionic strength on the RhB adsorption capacities of PCAC. Conditions: adsorbent, 20 mg; solution volume, 200 mL; initial concentration, 400 mg L^−1^; Temperature, 30 °C; pH, 3.

Usually, there are salts in dye wastewater, which may affect the adsorption of adsorbents toward dyes. Herein, the effect of ionic strength on the RhB adsorption of PCAC was investigated, when the pH value was 3. As shown in Figure 8, after sodium chloride (NaCl) was added into the RhB solution, the RhB adsorption capacity of PCAC slightly increased, which may be due to fact that an increase in ionic strength can weaken the electrostatic repulsion between RhB and PCAC [63]. However, in general, there were no obvious changes in RhB adsorption capacities along with the increment of ion strength, which is in agreement with the results of previous reports on the adsorption of RhB on various adsorbents [64,65,66]. This indicates that the electrostatic force between RhB and PCAC was weak [67].

#### 2.3.6. Reusability

**Figure 9:**
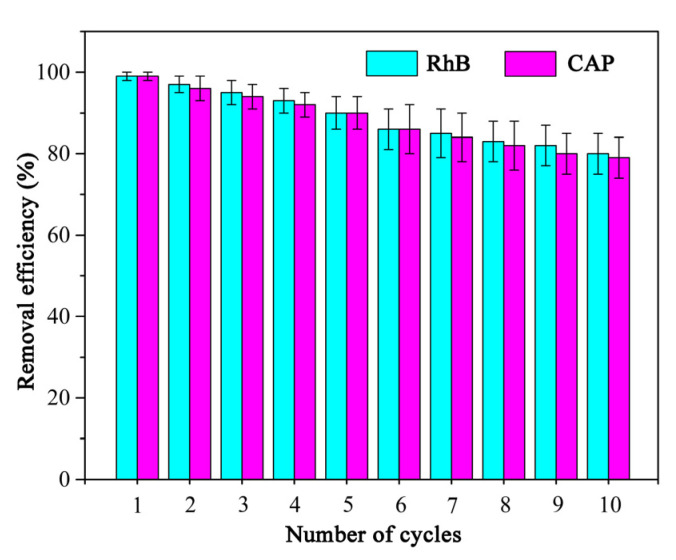
Reuse of PCAC for the adsorption of RhB and CAP. Conditions: adsorbent, 100 mg; solution volume, 100 mL; initial concentration, 50 mg L^−1^; Temperature, 30 °C. The pH values of RhB solutions were not adjusted.

The reusability of PCAC in the adsorption applications was evaluated through ten successive adsorption cycles (Figure 9). For avoiding potential secondary pollution, after the adsorbent was used, it was heated under N_2_ atmosphere for achieving the carbonization of adsorbates, which had been adsorbed by PCAC. After 10 cycles, the adsorption capacities of PCAC toward RhB and CAP decreased from 99% to approximately 80%, which indicates the good regenerative ability of PCAC.

### 2.4. Fixed-Bed Adsorption Column Study

**Figure 10:**
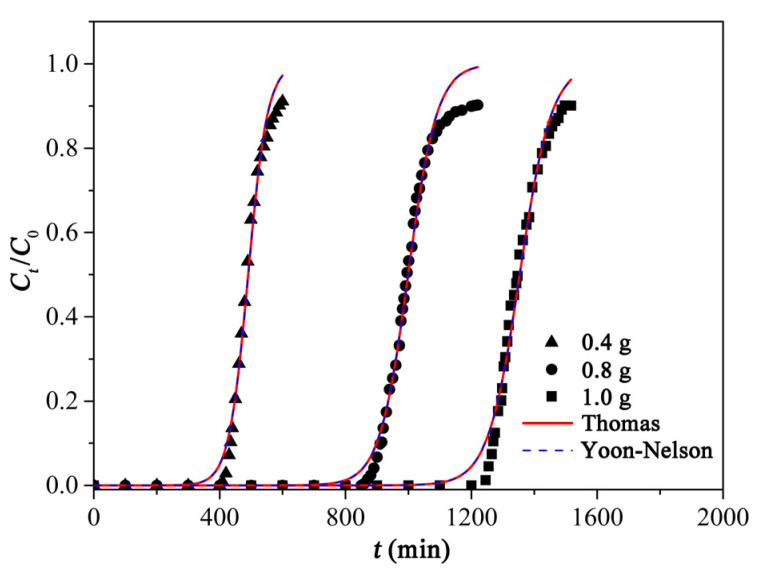
Breakthrough curves of RhB adsorption on PCAC with mass of 0.4 g, 0.8 g, and 1.0 g. The experimental data fit with the nonlinear Thomas model and the Yoon–Nelson model.

A series of fixed-bed adsorption experiments were carried out for investigating the effect of the dosages of adsorbent on the breakthrough curves. The breakthrough curves at a different mass of PCAC (0.4, 0.8, and 1.0 g) were shown in Figure 10, and the corresponding results were summarized in Table 5. When the permissible concentration of the effluent RhB solution reached 10% and 90% of the initial concentration, the times were called the breakthrough time (*t_b_*) and saturation time (*t_s_*), respectively. When the mass of PCAC increased from 0.4 to 1.0 g, *t_b_* increased from 435 to 1270 min, *t_s_* increased from 590 to 1497 min, and the volumes of treated RhB solutions increased from 3.2 to 8.2 L. When the mass of PCAC was 1.0 g, the value of ratio of the bed height to the inner diameter of column (*H*/*D*) was higher than 5, which is recommended for large-scale experiments because the maldistribution of adsorbate solutions in the adsorption processes may be avoided [68].

In the field of wastewater treatment, the Thomas and the Yoon–Nelson models were frequently applied to fit the experimental breakthrough curves. Herein, these adsorption kinetic models were used to investigate the adsorption of PCAC to RhB. The parameters of these models were calculated based on Equations (16) and (17) and shown in Table 6. When the masses of PCAC were 0.4, 0.8, and 1.0 g, respectively, the *R*^2^ values calculated based on these models were coincident. This is due to the fact that the Thomas and Yoon–Nelson models are mathematically the same [69]. The *R*^2^ values calculated based on these models were all higher than 0.98, which demonstrates that they fit the experimental data well.

Along with the increment in the adsorbent dosage, the fractional bed utilization (*FBU*) values gradually increased from 87.8 to 93.3%. Based on the result of the Yoon–Nelson model, the slopes (*k*) of breakthrough curves decreased from 0.032 to 0.020, and *τ* increased from 491 to 1354 min. These results showed that the dosage of adsorbent strongly affected the adsorption process, and the sample with more mass (or a higher bed height) was saturated more slowly than that with less mass (or a lower bed height). The higher dosage allowed more time for the interaction between PCAC and the adsorbate, which enabled the adsorbate molecules to diffuse into the deeper pores of PCAC, achieving better utilization of the adsorbent.

## 3. Materials and Methods

### 3.1. Materials

AC was obtained from the experimental field of Jilin Agricultural University, China. Deionized water was homemade in the laboratory. All the reagents were analytical pure grade and used without further purification. KOH, NaOH, and NaCl were purchased from Sinopharm Chemical Reagent Co., Ltd. (Shanghai, China). RhB and CAP were obtained from Aladdin Reagent Co., Ltd. (Shanghai, China).

### 3.2. Preparation of Porous Carbon

The sample of AC was washed with water, dried at 80 °C, and smashed with a pulverizer. The sample was filtered through a standard sieve (80 mesh). Then, AC was carbonized via being heated in a tube furnace at 500 °C for 60 min under N_2_. The produced biochar was further chemical activated with KOH and NaOH used as the activators, respectively. Typically, KOH was mixed with the biochar with the mass ratio of 4:1 in a mortar. The mixture was placed in a horizontal tubular furnace and heated under N_2_ at 800 °C for 60 min. After cooling to room temperature, the sample was taken out and washed with an HCl (1 M) aqueous solution and water until the pH of the filtrate became neutral. Finally, the porous carbon product was dried at 80 °C for 24 h and stored in a desiccator.

### 3.3. Characterization

The Zeiss Merlin scanning electron microscopy (SEM, Zeiss, Jena, Germany) was used for characterizing the surface morphologies of samples. N_2_ adsorption/desorption isotherms were measured at liquid nitrogen temperature using a Quantachrome Autosorb-iQ gas sorption analyzer (Quantachrome, Boynton Beach, FL, USA). Before the measurements, the porous carbon sample with a mass of approximately 100 mg was pretreated at 150 °C for 4 h under vacuum condition. A Nicolet iS5 FT-IR spectrophotometer (Thermo Fisher Scientific, Waltham, MA, USA) was used for recording the IR spectra. An Axis Ultra DLD spectrometer (Kratos Analytical, Manchester, UK) was applied for performing the XPS measurements. A LabRAM HR Evolution instrument (Horiba, Longjumeau, France) was applied for measuring the Raman spectra. The optical adsorptions of RhB and CAP solutions were measured on an Agilent Cary60 UV-Vis spectrophotometer (Agilent Technologies, Santa Clara, CA, USA). Element analysis of C, H, N, S, and O was performed on a vario EL Cube elemental analyzer (Elementar Analysensysteme GmbH, Langenselbold, Germany).

### 3.4. Batch Adsorption Experiments

Typically, the PCAC samples (20 mg) were added into the aqueous solutions (200 mL) of RhB (or CAP) with initial concentrations of 100, 200, 300, and 400 mg L^−^^1^, respectively. The pH values of solutions were not adjusted. The conical flasks containing the turbid liquids were placed in an air bath shaker with a shaking speed of 160 rpm at 30 °C. At pre-determined time, the liquids (1 mL) were collected and centrifuged at 10,000 rpm. The supernatants were analyzed with a UV-Vis spectrophotometer at 554 nm for RhB and 277 nm for CAP, respectively.

The equilibrium adsorption capacity of PCAC toward RhB (or CAP), *q_e_* (mg g^−^^1^), was calculated as follows (Equation (1)):

(1) $$q_{e}=\frac{\left(C_{0}-Ce\right)\times V}{m}$$

where *C*_0_ (mg L^−1^) and *C_e_* (mg L^−1^) are the initial and equilibrium concentrations of adsorbate solutions, respectively; *V* (L) is the volume of solution; and *m* (g) is the mass of PCAC.

### 3.5. Adsorption Kinetic Experiments

The effect of contact time on the adsorption capacity of PCAC toward RhB (or CAP) was investigated through the pseudo-first-order, pseudo-second-order, and intra-particle diffusion kinetic models (Equations (2)–(4)) [70].

(2) $$q_{t}=q_{e}(1-e^{-k_{1}t})$$

(3) $$q_{t}=\frac{k_{2}q_{e}^{2}}{\left(1+k_{2}q_{e}t\right)}t$$

(4) $$q_{t}=k_{i}t^{0.5}+B$$

where *q_t_* (mg g^−1^) is the RhB (or CAP) adsorption capacity of PCAC at contact time *t* (min); *k*_1_ (min^−1^), *k*_2_ (g mg^−1^ min^−1^), and *k_i_* (mg g^−1^ min^−1/2^) represent the rate constants of the pseudo-first-order, pseudo-second-order, and intra-particle diffusion kinetic models, respectively; and *B* is the parameter of the intra-particle diffusion model.

### 3.6. Adsorption Isotherm Experiments

In order to investigate the effect of the initial concentration of RhB (or CAP) on the adsorption capacities of PCAC, two non-linear adsorption isotherm models (Langmuir and Freundlich models (Equations (5) and (6)) with two parameters and two non-linear adsorption isotherm models (Sips and R-P models (Equations (7) and (8)) with three parameters were applied [71].

(5) $$q_{e}=\frac{q_{m}K_{L}C_{e}}{1+K_{L}C_{e}}$$

(6) $$q_{e}=K_{F}C_{e}^{1/n_{F}}$$

(7) $$q_{e}=\frac{q_{m}K_{s}C_{e}^{n_{s}}}{1+K_{s}C_{e}^{n_{s}}}$$

(8) $$q_{e}=\frac{K_{RP}C_{e}}{1+\alpha_{RP}C_{e}^{\beta }}$$

where *q_m_* (mg g^−1^) represents the maximum adsorption capacity of PCAC toward RhB (or CAP); *K_L_* (L mg^−1^) is the equilibrium constant of the Langmuir model; *K_F_* (mg g^−1^(L mg^−1^)^1/*n*^) and *n_F_* are the equilibrium constant and the heterogeneity factor of the Freundlich model, respectively; *K_s_* ((mg L^−1^)^−*n*^) and *n_s_* are the equilibrium constant and parameter of the Sips model, respectively; *K_RP_* (L mg^−1^), *α_RP_*, and *β* are the equilibrium constant and parameters of the R-P model, respectively.

### 3.7. Adsorption Thermodynamic Experiments

The adsorption experiments of RhB on PCAC were carried out at 30, 40, and 50 °C, respectively. The pH values of solutions were not adjusted. The standard entropy change (Δ*S*, J mol^−1^ K^−1^), the heat of the adsorption (Δ*H*, kJ mol^−1^), and the Gibbs free energy of adsorption (Δ*G*, kJ mol^−1^) were measured based on Equations (9)–(11) [72].

(9) ΔG=ΔH−TΔS

(10) $$\ln(K_{d})=\frac{\Delta S}{R}-\frac{\Delta H}{RT}$$

(11) $$K_{d}=q_{e}/C_{e}$$

where *K_d_* is the distribution coefficient; *T* (K) is temperature; and *R* (J K mol^−1^) is 8.314.

### 3.8. Reusability Studies

In each cycle, the adsorbent (100 mg) was added to the RhB or the CAP solution (50 mg L^−1^, 100 mL). The pH values of solutions were not adjusted. After recovery, the sample was heated at 500 °C for 60 min under N_2_ atmosphere in a tube furnace for carbonizing the adsorbates adsorbed by PCAC. Then, the carbonized sample was used as the adsorbent in the next adsorption cycle.

### 3.9. The Point of Zero Charge (PZC) of PCAC

The pH_PZC_ of PCAC was confirmed for characterizing the surface charge. A series of NaCl aqueous solutions (0.01 M) with pH values in the range of 3–11 were prepared. The pH values of solutions were adjusted through adding an HCl (0.1 M) or an NaOH (0.1 M) aqueous solution. The samples of PCAC (0.1 g) were added into the NaCl aqueous solutions (50 mL) with different pH values. The pH_PZC_ was determined by the intersection of the curves of pH_final_ and pH_initial_ [73].

### 3.10. Fixed-Bed Adsorption Column Study

The fixed-bed adsorption experiments were performed in a glass tube with an inner diameter of 1.2 cm, which was filled with the PCAC samples. The initial concentration of RhB was 200 mg L^−1^, the pH value of the RhB solution was not adjusted, and the downward flow rate was 5.5 mL min^−1^. At predetermined time intervals, the concentrations of effluent RhB solutions were monitored using the UV-Vis spectrophotometer.

The corresponding RhB adsorption capacities on PCAC at *t_b_* and *t_s_* were expressed as *q_b_* and *q_s_*, respectively (Equations (12) and (13)).

(12) $$q_{b}=\frac{Q}{1000}\frac{C_{0}}{m}\int_{t=0}^{t=t_{b}}\left(1-\frac{C_{t}}{C_{0}}\right)dt$$

(13) $$q_{s}=\frac{Q}{1000}\frac{C_{0}}{m}\int_{t=0}^{t=t_{s}}\left(1-\frac{C_{t}}{C_{0}}\right)dt$$

where *C*_0_ (mg L^−1^) is the initial RhB concentration; *C_t_* (mg L^−1^) is the RhB concentration of effluent at time *t* (min); *Q* (mL min^−1^) is the flow rate of RhB solution; and *m* (g) is the mass of PCAC [74].

The treated effluent volume *V_t_* (L) was calculated based on the flow rate and the saturation time, and *FBU* was calculated using the *q_b_* and *q_s_* (Equations (14) and (15)).

(14) $$V_{t}=Qt_{s}$$

(15) $$FBU=\left(\frac{q_{b}}{q_{s}}\right)\times 100\%$$

Three non-linear kinetic models (Thomas and Yoon–Nelson models) (Equations (16) and (17)) were used to fit the breakthrough curves in the fixed-bed column experiments [75].

(16) $$\frac{C_{t}}{C_{0}}=\frac{1}{1+exp\left(k_{Th}q_{0}m/Q-k_{Th}C_{0}t\right)}$$

(17) $$\frac{C_{t}}{C_{0}}=\frac{1}{1+exp\left(k_{YN}\tau -k_{YN}t\right)}$$

where *k_Th_* (L min^−1^ mg^−1^) and *q*_0_ (mg g^−1^) are the rate constant and equilibrium adsorption capacity of the Thomas model; *m* (g) is the mass of adsorbent; *Q* (L min^−1^) is the flow rate of the RhB aqueous solution; *k_YN_* (min^−1^) is the rate constant of the Yoon–Nelson model and *τ* (min) is the time required 50% of RhB breakthrough.

### 3.11. Error Analysis

For determining how well the kinetics and isothermal models fit the experimental data, the correlation coefficient (*R*^2^) and the sum of the squares of the errors (*SSE*) were calculated based on the following formulas (Equations (18) and (19)):

(18) $$R^{2}=\frac{\sum_{i=1}^{n}{\left(q_{exp,i}-q_{ave}\right)}^{2}-\sum_{i=1}^{n}{\left(q_{exp,i}-q_{cal,i}\right)}^{2}}{\sum_{i=1}^{n}{\left(q_{exp,i}-q_{ave}\right)}^{2}}$$

(19) $$SSE=\sum_{i=1}^{n}{\left(q_{cal,i}-q_{exp,i}\right)}_{i}^{2}$$

where *q_exp,i_*, *q_ave_*, and *q_ca1,i_*, are the experimental, average, and theoretical adsorption capacities (mg g^−1^) of PCAC toward the adsorbates, respectively.

## 4. Conclusions

It is the first time that the AC was used as the precursor for preparing the porous carbon material with a large BET specific surface area and a high adsorption ability toward the representative dye and antibiotic. KOH was confirmed to be better than NaOH as the activator because the porous carbon product activated by KOH exhibited a larger specific surface area, more mesopores, and a higher adsorption capacity than the product activated by NaOH. The pseudo-second-order kinetic and Sips isotherm models fit the adsorption experimental data well, and the maximum adsorption capacities of PCAC toward RhB and CAP reached 1883.3 and 1375.3 mg g^−1^, respectively. PCAC exhibited good reusability. RhB and CAP were adsorbed on the surface of PCAC via the pore-filling, π-π interactions, and H-bond interactions, and the adsorption process were physisorption and spontaneous. Moreover, PCAC exhibit a high performance in the fixed-bed column experiments, which indicates that it has the potential to be used in some wastewater treatment devices such as chromatographic columns, household water filters, and portable water filters. In sum, AC is a promising precursor for preparing the hierarchical porous carbon material with the advantages of low-cost, large specific surface area, and high adsorption performance. In the future, we will prepare the AC-derived composite materials toward the applications of gas capture, catalysis, supercapacitor, and controlled drug release.

## Figures and Tables

**Table 1:** Parameters of adsorption isothermal models for RhB and CAP adsorption on PCAC.

Isotherm Models	Parameters	Adsorbates	
		RhB	CAP
Langmuir	*q_m_* (mg g^−1^)	1378.8	1397.0
	*K_L_* (L mg^−1^)	0.16	0.06
	*R* ^2^	0.9272	0.9988
	*SSE*	3232.34	58.80
Freundlich	*K_F_* (mg g^−1^ (L mg^−1^)^1/*n*^)	674.20	498.00
	*n_F_*	7.76	5.76
	*R* ^2^	0.9855	0.8705
	*SSE*	1924.38	6773.32
Sips	*q_m_* (mg g^−1^)	1883.3	1375.3
	*K_s_*	0.07	0.06
	*n_s_*	0.35	1.09
	*R* ^2^	0.9998	0.9998
	*SSE*	9.00	10.22
Redlich-Peterson	*K_RP_* (L g^−1^)	605.90	74.27
	*b_RP_* (L mg^−1^)^−1/*α*^	0.72	0.05
	*α*	0.91	1.02
	*R* ^2^	0.9992	0.9995
	*SSE*	32.55	22.26

**Table 2:** Maximum adsorption capacities of RhB on adsorbents.

Adsorbents	Adsorption Capacities (mg g^−1^)	Refs.
Magnetic *Forsythia suspensa* leaf powders	34.014	[45]
graphene oxide/silicalite-1 composites	56.55	[46]
Tannic acid functionalized graphene	201	[47]
Gelatin/activated carbon composite	256.41	[48]
Activated carbon derived from bagasse pith	263.85	[49]
Rice husk-based activated carbon	518.1	[50]
Fe_3_O_4_ magnetic nanoparticles-graphene oxide	714.3	[51]
Lotus leaf porous carbon	718.9	[52]
PCAC	1883.3	This work

**Table 3:** Maximum adsorption capacities of CAP on adsorbents.

Adsorbents	Adsorption Capacities (mg g^−1^)	Refs.
Metal–organic framework (PCN-222)	370	[53]
Cellulose nanofibril/graphene oxide hybrid aerogel	418.7	[54]
Porous carbon produced from peanut shell	423.7	[55]
Porous carbon sheets from potassium acetate	588.2	[56]
N-doped hierarchically porous carbon	742.4	[57]
3D hierarchical porous biochar aerogel	786.1	[58]
Porous carbon derived from Enteromorpha prolifera	892.86	[59]
Bovine bone-derived porous carbon	1240	[60]
PCAC	1375.3	This work

**Table 4:** Thermodynamic parameters for adsorption of PCAC toward RhB and CAP.

Adsorbates	Temperature (°C)	Δ*H* (kJ mol^−1^)	Δ*S* (J mol^−1^)	Δ*G* (kJ mol^−1^)
RhB	30	2.10	20.76	−4.19
	40			−4.40
	50			−4.61
CAP	30	−2.12	6.12	−3.98
	40			−4.04
	50			−4.10

**Table 5:** Experimental data of the column parameters (Diameter of column, 1.2 cm; flow rate, 5.5 mL min^−1^; initial RhB concentration, 200 mg L^−1^). The pH values of RhB solutions were not adjusted.

*m* (g)	*H* (cm)	*H*/*D*	*C*_0_/*C* = 0.1		*C*_0_/*C* = 0.9		*FBU* (%)	*V_t_* (L)
			*t_b_* (min)	*q_b_* (mg g^−1^)	*t_s_* (min)	*q_s_* (mg g^−1^)		
0.4	2.7	2.3	435	1193.1	590	1358.1	87.8	3.2
0.8	5.4	4.5	915	1254.6	1200	1389.2	90.3	6.6
1.0	6.7	5.6	1270	1395.4	1497	1495.2	93.3	8.2

**Table 6:** Parameters of the nonlinear Thomas and Yoon–Nelsons models.

*m* (g)	Thomas Model			Yoon–Nelson Model		
	*k_Th_* (L min^−1^ mg^−1^)	*q*_0_ (mg L^−1^)	*R* ^2^	*k_YN_* (min^−1^)	*τ* (min)	*R* ^2^
0.4	1.6 × 10^−4^	1348.8	0.9862	0.032	491	0.9862
0.8	1.1 × 10^−4^	1373.6	0.9873	0.022	999	0.9873
1.0	1.0 × 10^−4^	1489.1	0.9861	0.020	1354	0.9861

## Data Availability

The data presented in this study are available in this paper and Supplementary File.

## Supplementary Materials

The following supporting information can be downloaded at: https://www.mdpi.com/article/10.3390/ijms23116146/s1.
