# Supplementary material for: A Weed-Derived Hierarchical Porous Carbon with a Large Specific Surface Area for Efficient Dye and Antibiotic Removal
Source: Int J Mol Sci. 2022 May 30;23(11):6146. doi: 10.3390/ijms23116146 (PMC9181242; doi:10.3390/ijms23116146)
Supplement: Supplementary file 1 [file ijms-23-06146-s001.zip › ijms-1725567-supplementary.pdf]

Supporting information

A weed-derived hierarchical porous carbon with large specific surface area for efficient removal of dye and antibiotic

Dadong Liang [1,2], Xingyi Tian [1], Yupeng Zhang [1], Guanya Zhu [1], Qiang Gao [1,2], Junbo Liu [1,*] and Xiaoxiao Yu [2]

[1] College of Resource and Environmental Science, Jilin Agricultural University, Changchun 130118, China; liangdadong@jlau.edu.cn (D.L.); tianxingyi222@163.com (X.T.); zhangyupeng1213@163.com (Y.Z.); zhuguanyacyy99@163.com (G.Z.); gyt199962@163.com (Q.G.)

[2] Key Laboratory of Straw Comprehensive Utilization and Black Soil Conservation, The Ministry of Education, Jilin Agricultural University, Changchun 130118, China; yx8751@sina.com (X.Y.)

* Correspondence: liujb@mail.ccut.edu.cn; Tel.: +086-0431-84532995

Contents

S1. Mechanism of chemical activation

Figure S1. (a, b, c) SEM images and (b) particle diameter distribution of PCAC.

Figure S2. The (a) C1s and (b) O1s XPS spectra of PCAC.

Figure S3. (a) the initial pH and finial pH of NaCl aqueous solutions after PCAC samples were added. (b) Effect of pH on the adsorption capacities of PCAC for RhB.

Table S1. Parameters of adsorption kinetic models for RhB adsorption on PCAC.

Table S2. Parameters of isotherm models for CAP adsorption on PCAC.

S1. Mechanism of chemical activation

When the biochar is heated under N_2 atmosphere with the presence of KOH, the possible chemical reaction steps are listed as following:

$$6KOH + 2C \rightarrow 2K + 3H_2 \uparrow + 2K_2CO_3 \quad \text{(S1)}$$

$$2KOH \rightarrow K_2O + H_2O \quad \text{(S2)}$$

$$K_2CO_3 \rightarrow K_2O + CO_2 \uparrow \quad \text{(S3)}$$

$$K_2CO_3 + 2C \rightarrow 2K + 3CO \uparrow \quad \text{(S4)}$$

$$K_2O + C \rightarrow 2K + CO \uparrow \quad \text{(S5)}$$

When NaOH was replaced by KOH, the possible chemical reaction steps are listed as following:

$$6NaOH + 2C \rightarrow 2Na + 2Na_2CO_3 + 3H_2 \uparrow \quad \text{(S6)}$$

$$Na_2CO_3 \rightarrow Na_2O + CO_2 \uparrow \quad \text{(S7)}$$

$$CO_2 + C \rightarrow 2CO \quad \text{(S8)}$$

After the reaction of biochar and alkali, a large number of micro- and mesopores occur in the surface of biochar, and the specific surface area of biochar may largely increased.

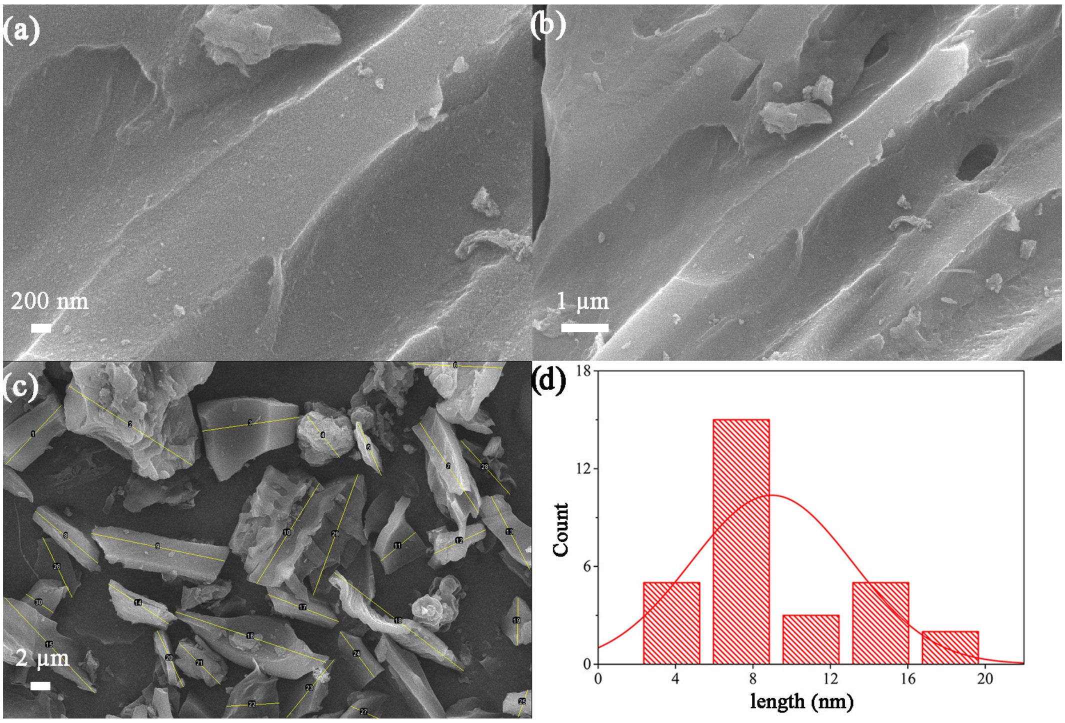

Figure S1. (a, b, c) SEM images and (b) particle size distribution of PCAC.

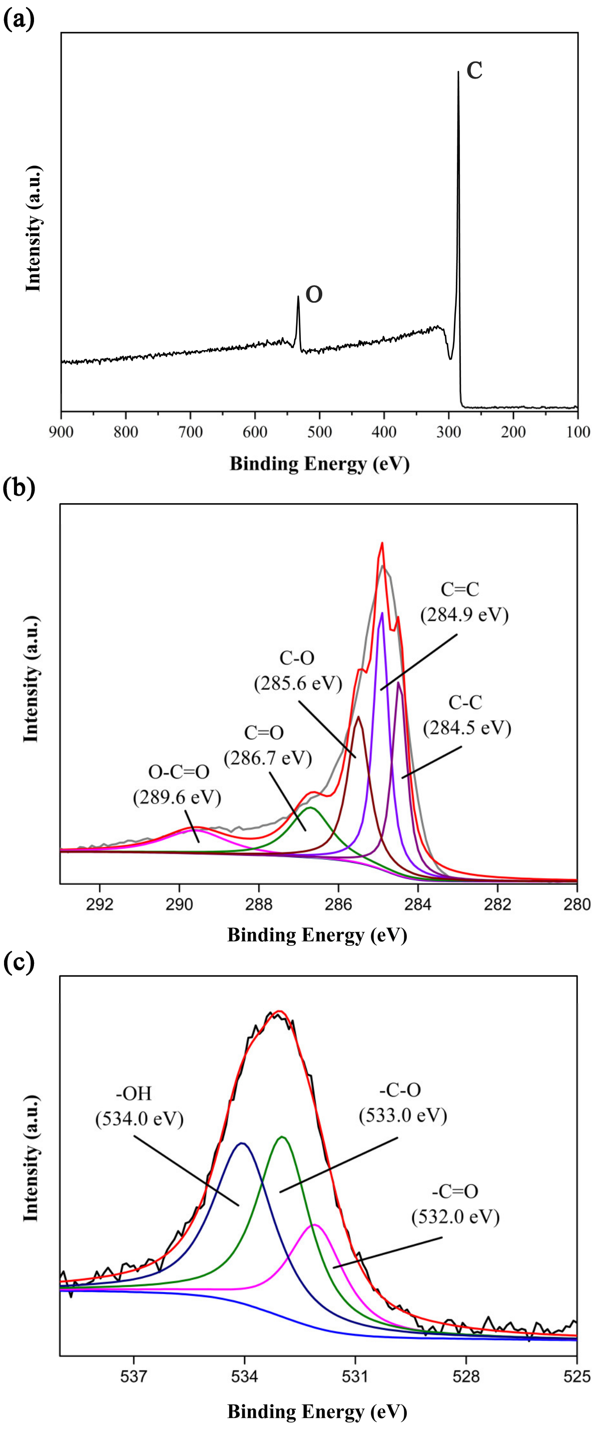

Figure S2. The XPS spectra of PCAC.

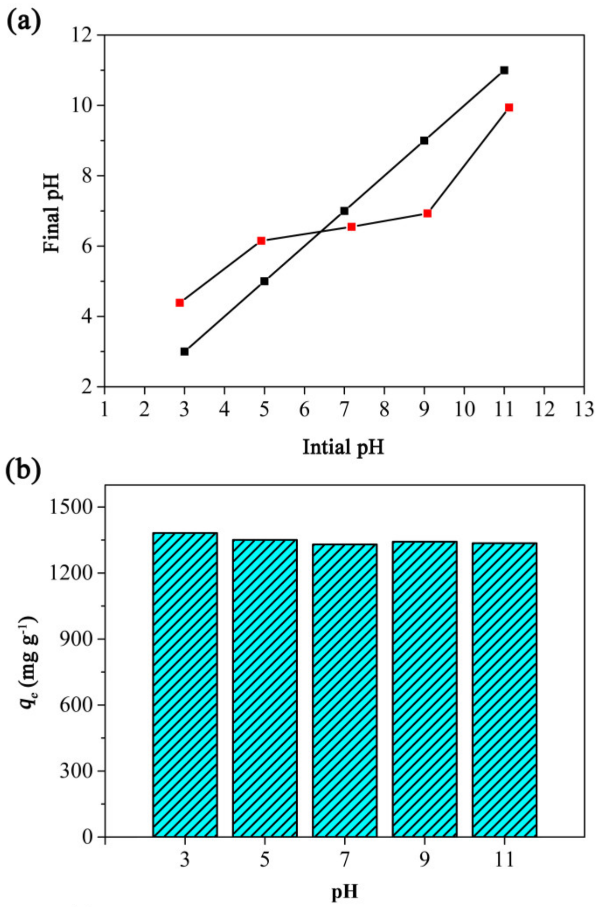

Figure S3. (a) the initial pH and finial pH of NaCl aqueous solutions after PCAC samples were added. (b) Effect of pH on the adsorption capacities of PCAC for RhB.

Table S1 Parameters of adsorption kinetic models for RhB adsorption on PCAC.

Models	Parameters	Initial concentration C_0 (mg L^{-1})			
		100	200	300	400
Pseudo-first-order	k_1 (min^{-1})	0.04	0.06	0.06	0.07
	q_e (mg g^{-1})	848.0	1134.6	1244.1	1315.3
	R^2	0.9837	0.9784	0.9773	0.9811
	SSE	1158.38	2683.36	3350.42	3085.31
Pseudo-second-order	k_2 (g mg^{-1} min^{-1})	6.2×10^{-5}	6.5×10^{-5}	6.9×10^{-5}	7.7×10^{-5}
	q_e (mg g^{-1})	958.4	1261.6	1372.2	1440.1
	R^2	0.9998	0.9998	0.9998	0.9998
	SSE	11.69	21.54	28.45	22.24
Intra-particle diffusion	$k_{i,1}$ (L g^{-1})	115.00	175.99	203.19	224.53
	B	-0.65	3.58	9.159	11.40
	R^2	0.9998	0.9985	0.9929	0.9910
	SSE	7.73	236.52	1550.78	2401.52
	$k_{i,2}$ (L g^{-1})	63.20	66.36	66.44	64.29
	B	268.50	537.29	658.71	762.36
	R^2	0.9778	0.9864	0.9680	0.9623
	SSE	171.41	115.24	275.07	304.33
	$k_{i,3}$ (L g^{-1})	14.01	17.24	14.95	10.61
	B	681.61	946.87	1093.48	1221.50
	R^2	0.9601	0.9854	0.9699	0.9097
	SSE	113.28	61.79	96.59	152.58

Table S2 Parameters of isotherm models for CAP adsorption on PCAC.

Models	Parameters	Initial concentration C_0 (mg L^{-1})			
		100	200	300	400
Pseudo-first-order	k_1 (min^{-1})	0.18	0.08	0.08	0.09
	q_e (mg g^{-1})	740.5	1110.7	1204.9	1235.3
	R^2	0.9830	0.9816	0.9818	0.97963
	SSE	860.00	2126.80	2482.34	2899.43
Pseudo-second-order	k_2 (g mg^{-1} min^{-1})	2.1×10^{-4}	1.0×10^{-4}	9.2×10^{-5}	1.0×10^{-4}
	q_e (mg g^{-1})	796.7	1211.5	1314.9	1339.2
	R^2	0.9990	0.9997	0.9998	0.9998
	SSE	1.83	28.35	17.78	18.63
Intra-particle diffusion	$k_{i,1}$ (L g^{-1})	140.92	196.02	211.98	225.76
	B	12.02	9.36	10.05	17.57
	R^2	0.9748	0.9920	0.99217	0.9790
	SSE	2672.67	1619.93	1866.43	5708.12
	$k_{i,2}$ (L g^{-1})	28.15	56.01	54.63	51.22
	B	510.74	642.83	737.29	805.39
	R^2	0.9896	0.9859	0.9706	0.9489
	SSE	15.82	85.03	170.42	264.52
	$k_{i,3}$ (L g^{-1})	4.75	8.57	9.65	9.95
	B	707.08	1036.78	1121.42	1152.76
	R^2	0.95097	0.8895	0.9914	0.9929
	SSE	14.96	123.56	11.34	9.86
